# A pathway analysis of genome-wide association study highlights novel type 2 diabetes risk pathways

**DOI:** 10.1038/s41598-017-12873-8

**Published:** 2017-10-02

**Authors:** Yang Liu, Jing Zhao, Tao Jiang, Mei Yu, Guohua Jiang, Yang Hu

**Affiliations:** 1 College of Basic Medical Sciences, Heilongjiang University of Chinese Medicine, Harbin, Heilongjiang China; 2 The Department of Obstetrics and Gynaecology, Heilongjiang Provincial Forestry General Hospital, Harbin, Heilongjiang China; 3 The 224th Hospital of Chinese People’s Liberation Army, Harbin, Heilongjiang China; 4 Research institute of Chinese Medicine in Heilongjiang province, Harbin, Heilongjiang China; 5 School of Life Science and Technology, Harbin Institute of Technology, Harbin, China

## Abstract

Genome-wide association studies (GWAS) have been widely used to identify common type 2 diabetes (T2D) variants. However, the known variants just explain less than 20% of the overall estimated genetic contribution to T2D. Pathway-based methods have been applied into T2D GWAS datasets to investigate the biological mechanisms and reported some novel T2D risk pathways. However, few pathways were shared in these studies. Here, we performed a pathway analysis using the summary results from a large-scale meta-analysis of T2D GWAS to investigate more genetic signals in T2D. Here, we selected PLNK and VEGAS to perform the gene-based test and WebGestalt to perform the pathway-based test. We identified 8 shared KEGG pathways after correction for multiple tests in both methods. We confirm previous findings, and highlight some new T2D risk pathways. We believe that our results may be helpful to study the genetic mechanisms of T2D.

## Introduction

Type 2 diabetes (T2D) is a common human complex disease caused by a combination of genetic and environmental factors^1^. T2D is characterized by a decrease in the number of functional insulin-producing β-cells^2^. Much effort has been devoted to identifying T2D susceptibility genes including linkage analysis, candidate gene study, and especially the genome-wide association studies (GWAS)^3^. However, the identified genetic variants or susceptibility genes just explain less than 20% of the overall estimated genetic contribution to T2D^4^. It is apparent that additional risk variants remain to be discovered.

Fortunately, several studies have demonstrated importance of pathway-based approaches^5–15^. Pathway-based methods have also been applied into T2D GWAS datasets to investigate the biological mechanisms. Perry *et al*. analyzed three T2D datasets from Diabetes Genetics Initiative (DGI), Wellcome Trust Case Control Consortium (WTCCC) and Finland-United States Investigation of Non-Insulin-Dependent Diabetes Mellitus Genetics (FUSION)^16^. Using a modified Gene Set Enrichment Algorithm (GSEA) algorithm, they reported 26 significant pathways including 6 KEGG (Kyoto Encyclopedia of Genes and Genomes) pathways, 3 BioCarta pathways, and 17 GO (Gene Ontology) pathways in the WTCCC dataset. The WNT signaling pathway was most strongly associated with T2D. However, no pathways were associated with T2D after correcting for multiple testing (*P* < 0.05). Zhong *et al*. developed a novel approach by integrating the pathway analysis and gene expression into T2D WTCCC GWAS dataset. They analyzed 110 KEGG pathways and identified 22 significant pathways (*P* < 0.05)^17^. However, only the olfactory transduction pathway and TGF-signaling pathway were shared in both studies^16,17^.

To investigate more genetic signals in T2D, we performed a pathway analysis using the summary results from a large-scale meta-analysis of T2D GWAS from the DIAGRAM Consortium (http://diagram-consortium.org/downloads.html)^18^. The meta-analysis consists of 12,171 T2D cases and 56,862 controls from 12 T2D GWAS from European descent populations^18^.

## Results

### Pathway analysis of T2D risk genes

Here, we first selected the PLNK^19^ and VEGAS^20^ software to perform the gene-based test, and the used the WebGestalt database to perform the pathway-based test. Both PLNK and VEGAS could adjust the gene length and LD patterns, and have been widely used. More detailed information is described in the Methods sections.

Using PLINK, we got 806 significant T2D risk genes with *P* < 0.05. Using WebGestalt, 731 of these 806 genes were mapped to 731 unique Entrez Gene IDs. We identified that these 731 genes were significantly enriched in 11 KEGG pathways with a Bonferroni correction test *P* < 0.05/220 = 2.27E-04 (supplementary Table 1). Metabolic pathways (hsa01100) is the most significant pathway with *P* = 4.92E-07. More detailed information about these 11 KEGG pathways is described in supplementary Table 1.

Using VEGAS, we got 1514 significant T2D risk genes with *P* < 0.05. Using WebGestalt, 1343 of these 1514 genes were mapped to 1514 unique Entrez Gene IDs. We further identified that these 1343 genes were significantly enriched in 44 KEGG pathways with a Bonferroni correction test *P* < 0.05/220 = 2.27E-04 (supplementary Table 2). Metabolic pathways (hsa01100) is the most significant pathway with *P* = 4.50E-10. More information about these 44 KEGG pathways is described in supplementary Table 2.

**Figure 1:**
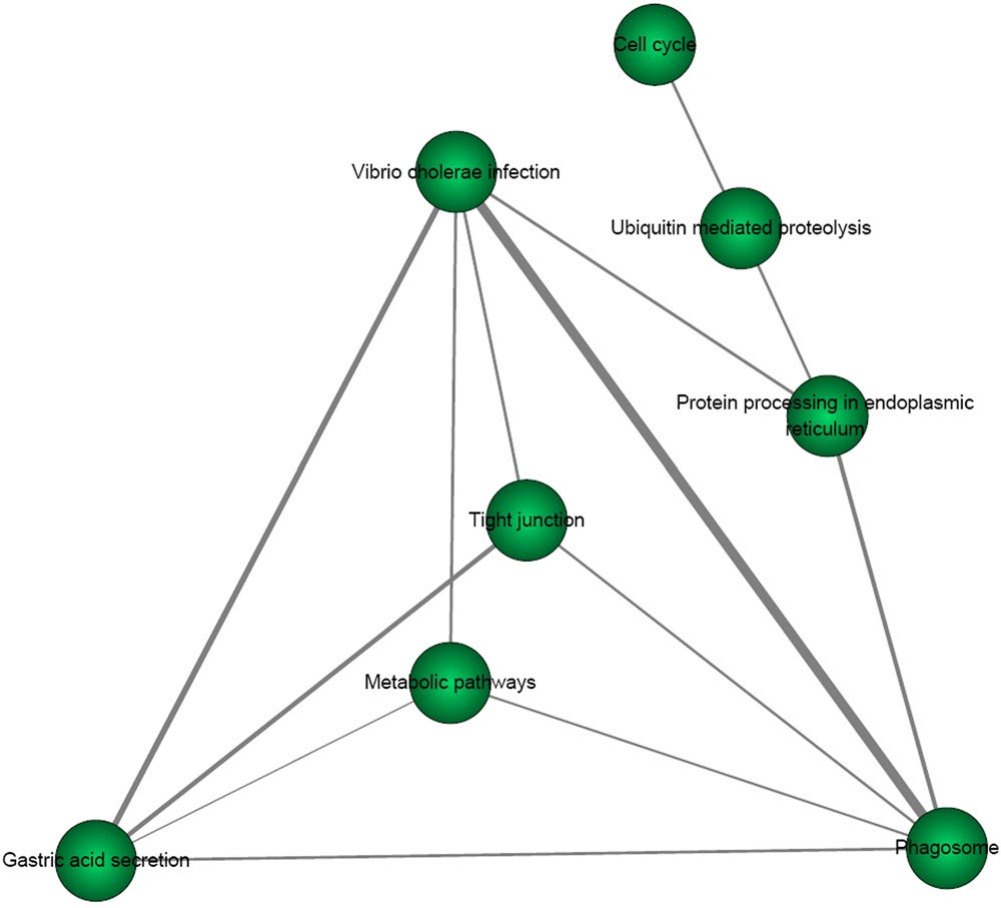
The pathway relationship of 8 KEGG pathways by pathway analysis of significant T2D risk genes from PLINK. The weight of pair-wise pathways is based on their related genes is defined by the Jaccard Index, given by the ratio of the intersection and union of the two gene sets.

**Figure 2:**
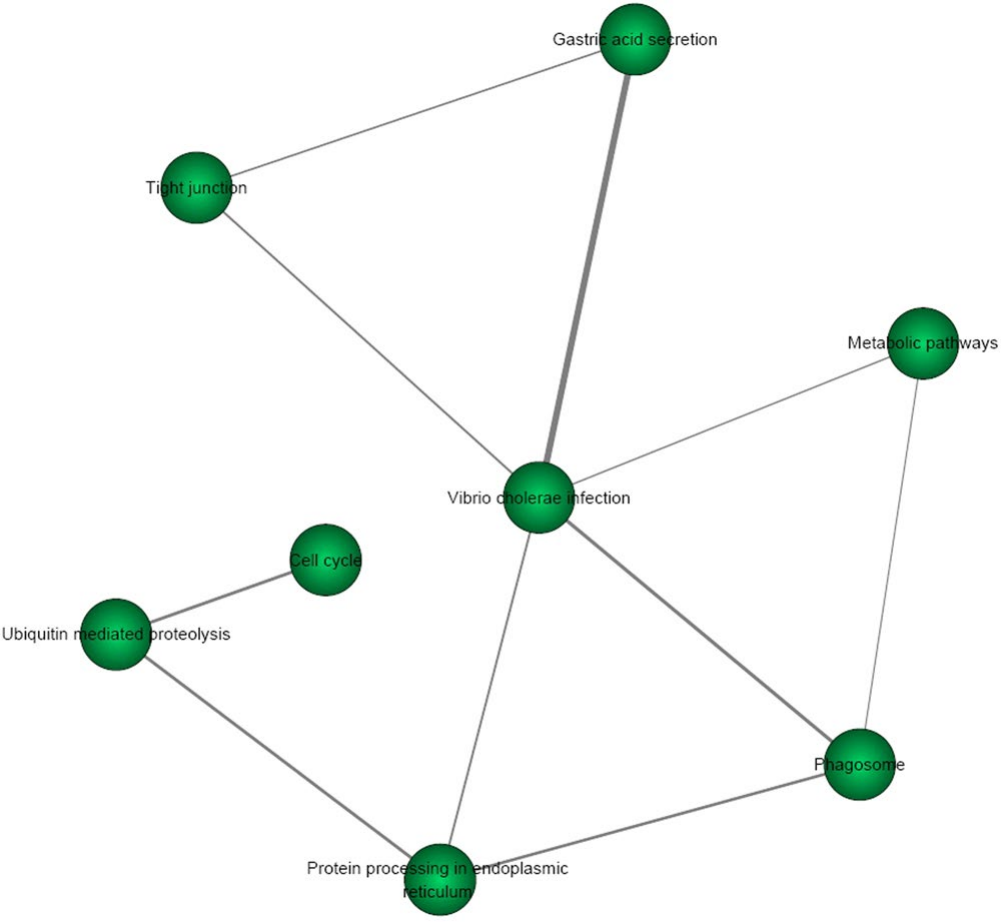
The pathway relationship of 8 KEGG pathways by pathway analysis of significant T2D risk genes from VEGAS. The weight of pair-wise pathways is based on their related genes is defined by the Jaccard Index, given by the ratio of the intersection and union of the two gene sets.

We further compared the T2D risk genes identified by the PLINK (n = 806) and VEGAS (n = 1514) methods. We found that 570 T2D risk genes were shared in these two methods. We also compared the pathway analysis results using T2D risk genes from PLINK and VEGAS. We identified 8 KEGG pathways to be shared in both methods with a Bonferroni correction test with *P* < 0.05/220 = 2.27E-04. Here, we list the results about the 8 shared pathways in Table 1. In order to get a better idea of these 8 pathways involved and how they correlated to each other, we provided pathway relationship in Fig. 1 and Fig. 2.

**Table 1:** 8 shared significant KEGG pathways with *P* < 2.27E-04 using genes from PLINK and VEGAS.

Pathway name	Enrichment analysis using risk genes from PLINK					Enrichment analysis using risk genes from VEGAS				
	C	O	E	R	*P* value	C	O	E	R	*P* value
Metabolic pathways	1130	48	19.15	2.51	8.48E-09	1130	81	35.06	2.31	3.91E-12
Tight junction	132	12	2.24	5.36	2.81E-06	132	17	4.1	4.15	7.72E-07
Vibrio cholerae infection	54	7	0.92	7.65	3.46E-05	54	8	1.68	4.78	2.00E-04
Gastric acid secretion	74	8	1.25	6.38	3.69E-05	74	10	2.3	4.36	9.46E-05
Cell cycle	124	10	2.1	4.76	5.30E-05	124	15	3.85	3.9	7.42E-06
Phagosome	153	11	2.59	4.24	6.53E-05	153	17	4.75	3.58	6.02E-06
Ubiquitin mediated proteolysis	135	10	2.29	4.37	1.00E-04	135	14	4.19	3.34	8.32E-05
Protein processing in endoplasmic reticulum	165	11	2.8	3.93	1.00E-04	165	15	5.12	2.93	2.00E-04

Abbreviations for all the six statistics in enrichment analysis: C, the number of reference genes in the category; O, the number of genes in the gene set and also in the category; E, expected number in the category; R, the ratio of enrichment,

### Comparison with previous pathway analysis

We further compared pathway analysis results with previous findings. Perry *et al*. identified a total of 26 pathways with a nominal *P* < 0.05 in WTCCC dataset including 6 KEGG pathways including WNT signaling pathway, Olfactory transduction, Galactose metabolism, Pyruvate metabolism, Type II diabetes, TGF-signaling pathway. Interestingly, we successfully replicated Type II diabetes as described in supplementary Table 2 ^16^.

Zhong *et al*. integrated the pathway analysis and gene expression into T2D WTCCC GWAS dataset^17^. They highlighted the involvement of 22 KEGG pathways in T2D risk^17^. Interestingly, we successfully replicated 9 of these 22 pathways including Tight junction, Neuroactive ligand-receptor interaction, Cell cycle, and Antigen processing and presentation, as described in supplementary Table 2. Meanwhile, both the Tight junction and Cell cycle are included in the 8 shared pathways with *P* < 0.05/220 = 2.27E-04.

## Discussion

Until now, pathway analysis of T2D GWAS dataset has been performed^16,17^. Here, we performed a pathway analysis of large-scale T2D GWAS meta-analysis dataset, and identified 9 significant pathways shared in both methods. We confirm previous findings, such as Tight junction and Cell cycle^17^. Here, we also highlight some new T2D risk pathways, such as Melanogenesis, Vibrio cholerae infection, Gastric acid secretion, Phagosome, Ubiquitin mediated proteolysis, and Protein processing in endoplasmic reticulum.

It is reported that pathway analysis of GWAS datasets may be more successful for some complex traits^21^. However, some pathway analysis methods may have limitations and the analysis results may be unstable^21^. The gene set size and gene length, LD patterns and the presence of overlapping genes could inflate the gene-score p-values and further may cause biases in pathway analysis^21,22^. It is suggested that multiple methods should be used to evaluate the reliability of the results^21^. Here, we selected PLNK^19^ and VEGAS^20^ to perform the gene-based test. Both PLNK and VEGAS could adjust the gene length and LD patterns.

There are some differences in PLINK and VEGAS. First, there are different statistic methods in PLINK and VEGAS. PLINK applied an approximate Fisher’s test to combine the *P* values across all the SNPs in genes^20^. In VEGAS, all the *P* values across all the SNPs in genes are converted to uppertail chi-squared statistics with one degree of freedom^20^. VEGAS applied the summarized chi-squared 1 degree of freedom statistics within a specific gene^20^. PLINK uses the average association test statistic across a given set of SNPs as the “set-based” test statistic^20^. VEGAS software uses the sum rather than average^20^. Second, there are different methods to map the SNPs to their corresponding genes in PLINK and VEGAS. In PLINK, a given set of SNPs were mapped to genes if these SNPs were located within the genomic sequence corresponding to the start of the first exon and the end of the last exon of any transcript corresponding to that gene^23^. In VEGAS, a given set of SNPs were mapped to ±50 kb of the 5′ and 3′ UTR of the corresponding genes^20^. Third, there are different methods to account for the LD, gene size, and the *P* value in PLINK and VEGAS. PLINK uses permutation test and VEGAS uses the simulation test^20^. Fourth, VEGAS method is much faster than the PLINK set-based test^20^. Here, we focus on the overlapping pathways resulting from the genes from PLINK and VEGAS methods based on these differences above.

Until now, two pathway analyses have been performed^16,17^. Perry *et al*. identified a total of 26 pathways^16^. Perry *et al*. analyzed 439 pathways from the Gene Ontology, BioCarta, and KEGG databases^16^. Meanwhile, Perry *et al*. selected the Bonferroni-adjusted *P* value to define the significant pathways, which further limits the number of significant pathways. Zhong *et al*. integrated the pathway analysis and gene expression into T2D WTCCC GWAS dataset^17^. They highlighted the involvement of 22 KEGG pathways in T2D risk^17^. We think that the integration of pathways and gene expression may be more powerful compared with the single pathway analysis, which may further reduce the number of significant pathways.

In summary, we analyzed the large-scale T2D GWAS meta-analysis dataset using PLNK and VEGAS. We not only confirm previous findings, but also highlight some new T2D risk pathways. We believe that our results may be helpful to study the genetic mechanisms of T2D. We will further replicate our findings using other available pathway analysis methods in future. Further replication studies are also required to evaluate our findings.

## Materials and Methods

### The T2D GWAS dataset

The GWAS dataset came from a meta-analysis of T2D GWAS datasets from DIAGRAM Consortium (http://diagram-consortium.org/downloads.html)^18^. Here, we give a brief description about the GWAS meta-analysis dataset^18^. More detailed information is provided in the original studies^18^. The meta-analysis consists of 12,171 T2D cases and 56,862 controls from 12 T2D GWAS from European descent populations^18^. All these samples were genotyped using kinds of genotyping platforms^18^. Each SNP was tested for association with T2D under an additive model after adjustment for study-specific covariates including indicators of population structure^18^. Full details of genotyping, quality control and imputation in each study are described in Supplementary Table 1 of the original study^18^. We got the association summary statistics from the original study^18^.

### Gene-based test using PLINK

PLINK (SET SCREEN TEST) was used to perform the gene-based test of the T2D GWAS dataset in the gene level^19^. This method is based on the meta-analysis of all the SNPs in genes using the linkage disequilibrium (LD) information from the HapMap CEU population^23^. PLINK applied an approximate Fisher’s test to combine *P* values across all the SNPs in genes to get the overall significance^23^. Meanwhile, PLINK supports larger genes (genes with more SNPs), and uses permutation testing to account for the correlation between SNPs (LD), gene size, and the *P* value from gene based test^23^.

### Gene-based test using VEGAS

VEGAS was also applied to conduct a gene-based test of the T2D GWAS dataset in the gene level^20^. The software utilizes all SNPs within a gene and adjusts the gene sizes, SNP density, and LD relation in SNPs^20^. VEGAS first assigns SNPs within ±50 kb from the 5′ and 3′ UTR to the corresponding genes according to the position information^20^. In a given gene, all the SNPs association *P* values are converted to uppertail chi-squared statistics with one degree of freedom^20^. The gene-based test statistic is the summarized chi-squared 1 degree of freedom statistics within a specific gene^20^. Meanwhile, simulations are used to adjust the LD relation in SNPs within a specific gene using the HapMap2 CEU genotype dataset^20^.

### Pathway-based test for T2D GWAS

We used the online database WebGestalt to conduct a pathway analysis^24^. In a specific KEGG pathway, a hypergeometric test was used to detect an overrepresentation of the T2D-related genes among all the genes in the pathway^24^. The entire Entrez gene set is selected to be the reference gene list. In a specific pathway, the minimum number of genes was 5. Meanwhile, a Bonferroni correction method was used to adjust for multiple tests with *P* < 0.05/220 = 2.27E-04. 220 is the number of KEGG pathways. A specific pathway with a *P* < 0.05/220 = 2.27E-04 is considered to be a significant pathway.

The weight of pair-wise pathways based on their related genes is defined on account of Jaccard Index as following.

1 $$weight({p}_{1},{p}_{2})=\frac{|{G}_{1}\cap {G}_{2}|}{|{G}_{1}\cup {G}_{2}|}$$

where *G*_*1*_ *and G*_*2*_ are the gene sets of pathway *p*_*1*_ *and p*_*2*_, respectively. |.| is the number of genes in the specified set. We then calculated the weights of all the pair-wise pathways and constructed a pathway network, where a node represents as a pathway and an edge as the weight of the pair-wise pathways more than zero.

## Electronic supplementary material

Dataset 1

## References

[1] Doria A, Patti ME, Kahn CR (2008). The emerging genetic architecture of type 2 diabetes. Cell Metab.

[2] Donath MY (2005). Mechanisms of beta-cell death in type 2 diabetes. Diabetes.

[3] Tsai FJ (2010). A genome-wide association study identifies susceptibility variants for type 2 diabetes in Han Chinese. PLoS Genet.

[4] Prasad RB, Groop L (2015). Genetics of type 2 diabetes-pitfalls and possibilities. Genes (Basel).

[5] Wang K, Li M, Hakonarson H (2010). Analysing biological pathways in genome-wide association studies. Nat Rev Genet.

[6] Wei J (2016). Multiple analyses of large-scale genome-wide association study highlight new risk pathways in lumbar spine bone mineral density. Oncotarget.

[7] Bao X (2015). Cell adhesion molecule pathway genes are regulated by cis-regulatory SNPs and show significantly altered expression in Alzheimer’s disease brains. Neurobiol Aging.

[8] Zhao X (2015). Pathway analysis of body mass index genome-wide association study highlights risk pathways in cardiovascular disease. Sci Rep.

[9] Xiang Z (2015). Integrating Genome-Wide Association Study and Brain Expression Data Highlights Cell Adhesion Molecules and Purine Metabolism in Alzheimer’s Disease. Mol Neurobiol.

[10] Quan B (2015). Pathway analysis of genome-wide association study and transcriptome data highlights new biological pathways in colorectal cancer. Mol Genet Genomics.

[11] Liu G (2014). Cardiovascular disease contributes to Alzheimer’s disease: evidence from large-scale genome-wide association studies. Neurobiol Aging.

[12] Liu G (2013). Measles contributes to rheumatoid arthritis: evidence from pathway and network analyses of genome-wide association studies. PLoS One.

[13] Liu G (2012). Cell adhesion molecules contribute to Alzheimer’s disease: multiple pathway analyses of two genome-wide association studies. J Neurochem.

[14] Jiang Q (2017). Alzheimer’s Disease Variants with the Genome-Wide Significance are Significantly Enriched in Immune Pathways and Active in Immune Cells. Mol Neurobiol.

[15] Liu G (2017). Integrating genome-wide association studies and gene expression data highlights dysregulated multiple sclerosis risk pathways. Mult Scler.

[16] Perry JR (2009). Interrogating type 2 diabetes genome-wide association data using a biological pathway-based approach. Diabetes.

[17] Zhong H, Yang X, Kaplan LM, Molony C, Schadt EE (2010). Integrating pathway analysis and genetics of gene expression for genome-wide association studies. Am J Hum Genet.

[18] Morris AP (2012). Large-scale association analysis provides insights into the genetic architecture and pathophysiology of type 2 diabetes. Nat Genet.

[19] Purcell S (2007). PLINK: a tool set for whole-genome association and population-based linkage analyses. Am J Hum Genet.

[20] Liu JZ (2010). A versatile gene-based test for genome-wide association studies. Am J Hum Genet.

[21] Jia P, Wang L, Meltzer HY, Zhao Z (2011). Pathway-based analysis of GWAS datasets: effective but caution required. Int J Neuropsychopharmacol.

[22] Wang, L., Jia, P., Wolfinger, R. D., Chen, X. & Zhao, Z. Gene set analysis of genome-wide association studies: Methodological issues and perspectives. *Genomics* (2011). 10.1016/j.ygeno.2011.04.006 PMC3852939 21565265

[23] Moskvina V (2011). Evaluation of an approximation method for assessment of overall significance of multiple-dependent tests in a genomewide association study. Genet Epidemiol.

[24] Wang J, Duncan D, Shi Z, Zhang B (2013). WEB-based GEne SeT AnaLysis Toolkit (WebGestalt): update 2013. Nucleic Acids Res.

