# Supplementary material for: A pathway analysis of genome-wide association study highlights novel type 2 diabetes risk pathways
Source: Sci Rep. 2017 Oct 2;7:12546. doi: 10.1038/s41598-017-12873-8 (PMC5624908; doi:10.1038/s41598-017-12873-8)
Supplement: Supplementary file 1 — Dataset 1 [file 41598_2017_12873_MOESM1_ESM.doc]

A pathway analysis of genome-wide association study highlights novel type 2 diabetes risk pathways

Yang Liu1, [[1]](#endnote-2)*, Jing Zhao^2^, Tao Jiang^3^, Mei Yu^4^, Guohua Jiang^1^ , Yang Hu5, [[2]](#endnote-3)*

^1^College of Basic Medical Sciences, Heilongjiang University of Chinese Medicine, Harbin, Heilongjiang, China

^2^The Department of Obstetrics and Gynaecology, Heilongjiang Provincial Forestry General Hospital, Harbin Heilongjiang, China

^3^The 224th Hospital of Chinese People’s Liberation Army, Harbin, Heilongjiang, China

^4^Research institute of Chinese Medicine in Heilongjiang province, Harbin, Heilongjiang, China

^5^School of Life Science and Technology, Harbin Institute of Technology, Harbin, China

*Corresponding author: Yang Liu

College of Basic Medical Sciences, Heilongjiang University of Chinese Medicine, Harbin, Heilongjiang, China

E-mail: [yangliu_hlj@126.com](mailto:yangliu_hlj@126.com)

*Corresponding author: Yang Hu

School of Life Science and Technology, Harbin Institute of Technology, Room 417, Building 2E, Science Park, Yikuang Street, Nangang District, Harbin 150080, China

E-mail: [huyang@hit.edu.cn](mailto:huyang@hit.edu.cn)

Supplementary Table 1, detailed information about 11 KEGG pathways using genes identified by PLINK

| Pathway name | ID | *P* value | Significant T2D genes |
| --- | --- | --- | --- |
| Metabolic pathways | hsa01100 | 8.48E-09 | PPT2 ACADL EHHADH GCDH INPP1 ARG1 ATP5J PLCB3 AMY2B PFKL ATP6V1G2 QDPR NDUFB2 HIBCH DAO APIP DGAT1 PNPLA3 ACAD8 HSD17B12 IMPA2 ALG10B PIGY ALG1 ATP5G1 AGXT GAPDH NDUFC1 LAP3 GCLC PDHA2 POLG2 PDHX MAN2A2 POLR2C PLCG1 REV3L ACADSB GCK PAFAH2 GPT EXTL1 PTGDS ALAS1 GALNT10 G6PC2 NDST3 ATP6V0E1 |
| Huntington's disease | hsa05016 | 6.30E-07 | CREB3L2 ATP5G1 POLR2C AP2M1 NDUFC1 PPARG NDUFB2 TP53 DLG4 VDAC3 CYCS TGM2 CREB3 ATP5J PLCB3 |
| Tight junction | hsa04530 | 2.81E-06 | IGSF5 MYH3 VAPA ACTB CSNK2B MRAS CLDN18 GNAI3 JAM2 PPP2R2D MYH1 CLDN7 |
| Melanogenesis | hsa04916 | 8.84E-06 | CREB3L2 ADCY5 MITF CREB3 GNAI3 CAMK2G TCF7L2 DVL2 PLCB3 ADCY7 |
| Gastric acid secretion | hsa04971 | 3.69E-05 | ACTB KCNQ1 KCNJ2 GNAI3 ADCY5 CAMK2G ADCY7 PLCB3 |
| Vibrio cholerae infection | hsa05110 | 3.46E-05 | ACTB PLCG1 KCNQ1 ATP6V0E1 ATP6V1G2 KDELR1 SEC61A2 |
| Cell cycle | hsa04110 | 5.30E-05 | CDKN2B CDKN2C CCNA2 BUB3 ANAPC13 E2F4 TFDP1 CCNA1 TP53 MCM3 |
| Phagosome | hsa04145 | 6.53E-05 | HLA-DRB1 CALR HLA-DRA ATP6V1G2 ITGA5 ACTB RAB5A HLA-C ATP6V0E1 SEC61A2 TUBA1B |
| Protein processing in endoplasmic reticulum | hsa04141 | 1.00E-04 | CALR UBE2D3 HSPA1L SEC24C TRAF2 WFS1 HYOU1 SSR2 TXNDC5 SSR1 SEC61A2 |
| Ubiquitin mediated proteolysis | hsa04120 | 1.00E-04 | UBE2D3 ANAPC13 HERC1 ITCH UBE2Z FBXW7 UBE2R2 TRIM37 SMURF1 SOCS3 |
| Viral myocarditis | hsa05416 | 2.00E-04 | HLA-DRB1 HLA-DRA CYCS ACTB HLA-C MYH1 MYH3 |

Supplementary Table 2, detailed information about 44 KEGG pathways using genes identified by VEGAS

| Pathway name | ID | *P* value | Significant T2D genes |
| --- | --- | --- | --- |
| Metabolic pathways | hsa01100 | 3.91E-12 | SRM ACADL PPT2 ACSL1 AGPAT1 GCDH ARG1 PLB1 ATP5J PIGL PNMT ATP6V1G2 G6PC SUCLG2 HIBCH APIP ALG10B ACADS AGXT DTYMK LAP3 POLG2 PDHX PNLIPRP3 AKR1A1 AGPAT6 MAN2A2 PLCG1 GCK AGPAT2 PAFAH2 BDH2 ALAS1 NDUFS3 GALNT10 PHOSPHO2 ST3GAL1 NDST3 CKM EHHADH CYP21A2 AGPAT9 BHMT AGPAT3 ISYNA1 PIGU INPP1 ST6GALNAC4 DPM2 AMY2B PNLIP NDUFB2 PTS ST8SIA1 DGAT1 ACAD8 PNPLA3 GMDS HSD17B12 IMPA2 FAH AMPD2 ATP5G1 NDST2 BCKDHB MTHFR GCLC RDH10 PDHA2 GCNT3 NT5C2 REV3L ACADSB EXTL1 DEGS1 MCAT PIP5KL1 ACADVL PTGES PANK3 POLD2 |
| Allograft rejection | hsa05330 | 1.00E-08 | TNF HLA-DRB1 HLA-DRA HLA-B HLA-DQA1 HLA-DQB1 HLA-E IL2 HLA-C HLA-DRB5 IL5 |
| Viral myocarditis | hsa05416 | 2.73E-08 | HLA-DRB1 HLA-DRA CCND1 HLA-B ITGAL HLA-DQA1 HLA-DQB1 HLA-E MYH3 HLA-C MYH14 MYH2 MYH1 HLA-DRB5 |
| Type I diabetes mellitus | hsa04940 | 5.68E-08 | TNF HLA-DRB1 HLA-DRA HLA-B HLA-DQA1 HLA-DQB1 HLA-E IL2 HLA-C LTA HLA-DRB5 |
| Toxoplasmosis | hsa05145 | 1.54E-07 | TNF PPIF HLA-DRA LAMA4 LAMC1 HLA-DQA1 CHUK LAMA1 GNAI3 HSPA1B HLA-DRB1 HLA-DQB1 NFKB1 PIK3R3 PIK3CD IFNGR1 STAT3 HLA-DRB5 |
| Type II diabetes mellitus | hsa04930 | 1.94E-07 | TNF ABCC8 IRS2 SLC2A4 GCK CACNA1G SLC2A2 PIK3R3 PIK3CD KCNJ11 ADIPOQ |
| Small cell lung cancer | hsa05222 | 3.48E-07 | CDKN2B CCNE2 LAMA4 CCND1 LAMC1 TRAF2 ITGA2 NFKB1 E2F3 PIK3R3 CHUK PIK3CD LAMA1 CDK6 |
| Graft-versus-host disease | hsa05332 | 3.73E-07 | TNF HLA-DRB1 HLA-DRA HLA-B HLA-DQA1 HLA-DQB1 HLA-E IL2 HLA-C HLA-DRB5 |
| Glycerolipid metabolism | hsa00561 | 3.03E-07 | AGPAT2 PNLIP AGPAT9 AGPAT1 LPL AGPAT3 PNPLA3 DGAT1 PNLIPRP3 AGPAT6 AKR1A1 |
| ECM-receptor interaction | hsa04512 | 3.48E-07 | LAMA4 SV2C LAMC1 SV2A ITGA7 ITGA2 COL6A1 COL2A1 TNXB SDC2 ITGA5 ITGA1 LAMA1 ITGB6 |
| Autoimmune thyroid disease | hsa05320 | 4.62E-07 | HLA-DRB1 HLA-DRA HLA-B HLA-DQA1 HLA-DQB1 HLA-E IL2 TG HLA-C HLA-DRB5 IL5 |
| Chemokine signaling pathway | hsa04062 | 5.08E-07 | BRAF GRK5 PREX1 CHUK CCL17 BCAR1 GNAI3 PRKACA CXCL9 CXCL10 CXCL11 CCR4 ADCY5 NFKB1 PIK3R3 PIK3CD ROCK1 STAT3 GRK1 PRKACB ADCY7 |
| Tight junction | hsa04530 | 7.72E-07 | TJP2 EPB41L3 MYL7 IGSF5 MYH3 VAPA MRAS CSNK2B MYH14 PPP2R2C CLDN18 GNAI3 JAM2 SYMPK MYH2 MYH1 CLDN7 |
| Focal adhesion | hsa04510 | 1.29E-06 | LAMA4 PDPK1 PDGFC BRAF MYL7 LAMC1 TLN1 TNXB ITGA5 BCAR1 LAMA1 CCND1 ITGA7 COL2A1 COL6A1 ITGA2 PIK3R3 PIK3CD ROCK1 ITGA1 ITGB6 |
| MAPK signaling pathway | hsa04010 | 1.21E-06 | TNF MAP3K3 BRAF MAP3K13 CACNA1G TRAF2 MRAS CHUK FOS NGF PRKACA RPS6KA5 MAPK8IP1 HSPA1B FGFR3 DUSP16 NFKB1 MAP3K11 CDC25B NFKB2 CACNA1I CACNG2 PRKACB DUSP1 MAP3K1 |
| Pathways in cancer | hsa05200 | 1.49E-06 | CDKN2B CCNE2 LAMA4 BRAF LAMC1 PPARG TRAF2 E2F3 DVL3 CHUK LAMA1 FOS TCF7L2 CCND1 FGFR3 PLCG1 EGLN2 ITGA2 MITF NFKB1 BAX PIK3R3 PIK3CD NFKB2 STAT3 CDK6 DVL2 CDKN2A |
| Antigen processing and presentation | hsa04612 | 3.76E-06 | TNF HLA-DRB1 HLA-DRA CALR HLA-B NFYA HLA-DQA1 HLA-DQB1 HLA-E HLA-C HLA-DRB5 HSPA1B |
| Dilated cardiomyopathy | hsa05414 | 4.17E-06 | TNF TNNC1 ADCY5 ITGA7 ITGA2 ITGA5 ITGA1 PRKACA ITGB6 CACNG2 PRKACB ADCY7 MYL3 |
| Cell adhesion molecules (CAMs) | hsa04514 | 4.01E-06 | HLA-DRA ITGAL HLA-DQA1 SELPLG HLA-E SDC2 NRXN1 CD58 HLA-DRB1 HLA-B HLA-DQB1 HLA-C CLDN18 JAM2 HLA-DRB5 CLDN7 |
| Neuroactive ligand-receptor interaction | hsa04080 | 5.16E-06 | P2RX4 MTNR1B PRSS3 NPBWR2 CYSLTR2 OPRM1 GIPR GLP2R PTGER3 CRHR2 CHRNA5 TSPO MC3R GRM4 OPRL1 CHRNA3 CCKAR TAAR1 P2RX5 GLRA1 TAAR2 HCRTR2 HRH1 CHRNB4 |
| Endocytosis | hsa04144 | 5.21E-06 | AP2M1 GRK5 EPS15 ITCH HLA-E USP8 SNF8 RAB5A VPS37C HSPA1B EPN3 HLA-B FGFR3 VPS28 PIP5KL1 PSD VPS4A HLA-C GRK1 SMURF1 |
| Amoebiasis | hsa05146 | 5.30E-06 | TNF LAMA4 C8G LAMC1 NFKB1 COL2A1 PIK3R3 MUC2 RAB5A PIK3CD LAMA1 ARG1 PRKACA PRKACB |
| Phagosome | hsa04145 | 6.02E-06 | HLA-DRB1 HLA-DRA CALR HLA-B MRC2 HLA-DQA1 HLA-DQB1 ATP6V1G2 HLA-E ITGA2 ITGA5 RAB5A HLA-C MPO HLA-DRB5 SEC61A2 TUBA1B |
| Staphylococcus aureus infection | hsa05150 | 6.56E-06 | HLA-DRB1 HLA-DRA ITGAL HLA-DQA1 HLA-DQB1 SELPLG CFB C2 HLA-DRB5 MASP2 |
| Adipocytokine signaling pathway | hsa04920 | 7.46E-06 | TNF IRS2 CAMKK2 ACSL1 SLC2A4 TRAF2 NFKB1 G6PC CHUK STAT3 ADIPOQ |
| Cell cycle | hsa04110 | 7.42E-06 | CDKN2B CCNE2 CCND1 CDKN2C BUB3 ANAPC13 CDKN1C TFDP1 E2F3 CDC25B RBL2 CDK6 ANAPC4 CDKN2A MCM3 |
| Prostate cancer | hsa05215 | 1.99E-05 | CREB3L2 CCNE2 PDPK1 CCND1 PDGFC BRAF NFKB1 E2F3 PIK3R3 CHUK PIK3CD TCF7L2 |
| Asthma | hsa05310 | 2.96E-05 | TNF HLA-DRB1 HLA-DRA HLA-DQA1 HLA-DQB1 HLA-DRB5 IL5 |
| Biosynthesis of unsaturated fatty acids | hsa01040 | 3.21E-05 | FADS1 ELOVL5 FADS2 ELOVL2 HSD17B12 PTPLA |
| Progesterone-mediated oocyte maturation | hsa04914 | 7.16E-05 | BRAF ANAPC13 ADCY5 PIK3R3 CDC25B PIK3CD GNAI3 PRKACA ANAPC4 PRKACB ADCY7 |
| Complement and coagulation cascades | hsa04610 | 5.15E-05 | C8G F13B PLAU SERPIND1 PROC CFB PROS1 C2 MASP2 F3 |
| Pancreatic cancer | hsa05212 | 5.84E-05 | CCND1 BRAF NFKB1 E2F3 PIK3R3 CHUK PIK3CD STAT3 CDK6 CDKN2A |
| Non-small cell lung cancer | hsa05223 | 3.92E-05 | PDPK1 CCND1 PLCG1 BRAF E2F3 PIK3R3 PIK3CD CDK6 CDKN2A |
| Protein digestion and absorption | hsa04974 | 4.08E-05 | CTRB1 CPA3 COL6A1 PRSS3 COL2A1 PGA3 PGA4 ATP1B2 KCNQ1 SLC16A10 CPB1 |
| Ubiquitin mediated proteolysis | hsa04120 | 8.32E-05 | UBE2D3 UBE2E2 ANAPC13 RHOBTB1 ITCH HERC1 UBE2Z FBXW7 UBE3C SMURF1 UBE2R2 TRIM37 ANAPC4 MAP3K1 |
| Gastric acid secretion | hsa04971 | 9.46E-05 | ADCY5 ATP1B2 KCNQ1 GNAI3 PRKACA ATP4B PRKACB CAMK2G ADCY7 KCNE2 |
| Chagas disease (American trypanosomiasis) | hsa05142 | 9.46E-05 | TNF CALR NFKB1 IL2 PIK3R3 CHUK PIK3CD TLR9 PPP2R2C IFNGR1 GNAI3 FOS |
| Apoptosis | hsa04210 | 7.97E-05 | TNF TRAF2 NFKB1 BAX PIK3R3 CHUK PIK3CD PRKACA NGF PRKACB PRKAR1A |
| Regulation of actin cytoskeleton | hsa04810 | 1.00E-04 | ITGAL PDGFC SSH2 BRAF MYL7 ITGA5 MRAS BCAR1 PIP4K2B FGFR3 ITGA7 ITGA2 PIK3R3 MYH14 PIK3CD ROCK1 ITGA1 ITGB6 |
| Neurotrophin signaling pathway | hsa04722 | 2.00E-04 | MAP3K3 IRS2 PLCG1 BRAF NFKB1 BAX PIK3R3 PIK3CD NGF RPS6KA5 GAB1 CAMK2G MAP3K1 |
| Vibrio cholerae infection | hsa05110 | 2.00E-04 | TJP2 PLCG1 ATP6V1G2 MUC2 KCNQ1 PRKACA PRKACB SEC61A2 |
| Osteoclast differentiation | hsa04380 | 2.00E-04 | TNF PPARG TRAF2 NFKB1 MITF FOSB PIK3R3 CHUK PIK3CD NFKB2 IFNGR1 NFATC1 FOS |
| Glioma | hsa05214 | 2.00E-04 | CCND1 PLCG1 BRAF E2F3 PIK3R3 PIK3CD CDK6 CAMK2G CDKN2A |
| Protein processing in endoplasmic reticulum | hsa04141 | 2.00E-04 | UBE2D3 CALR SEC13 UBE2E2 AMFR SEC24C TRAF2 WFS1 BAX RNF5 HSPA1B RAD23A SEC61A2 TXNDC5 SSR1 |

1. ^^ [↑](#endnote-ref-2)
2. ^^ [↑](#endnote-ref-3)
